# Inhibitory Effects of Salinomycin on Cell Survival, Colony Growth, Migration, and Invasion of Human Non-Small Cell Lung Cancer A549 and LNM35: Involvement of NAG-1

**DOI:** 10.1371/journal.pone.0066931

**Published:** 2013-06-21

**Authors:** Kholoud Arafat, Rabah Iratni, Takashi Takahashi, Khatija Parekh, Yusra Al Dhaheri, Thomas E. Adrian, Samir Attoub

**Affiliations:** 1 Department of Pharmacology and Therapeutics, College of Medicine and Health Sciences, United Arab Emirates University, Al-Ain, United Arab Emirates; 2 Department of Biology, College of Science, United Arab Emirates University, Al-Ain, United Arab Emirates; 3 Division of Molecular Carcinogenesis, Center for Neurological Diseases and Cancer, Nagoya University Graduate School of Medicine, Nagoya, Japan; 4 Departments of Physiology, College of Medicine and Health Sciences, United Arab Emirates University, Al-Ain, United Arab Emirates; University of Porto, Portugal

## Abstract

A major challenge for oncologists and pharmacologists is to develop more potent and less toxic drugs that will decrease the tumor growth and improve the survival of lung cancer patients. Salinomycin is a polyether antibiotic used to kill gram-positive bacteria including mycobacteria, protozoans such as plasmodium falciparum, and the parasites responsible for the poultry disease coccidiosis. This old agent is now a serious anti-cancer drug candidate that selectively inhibits the growth of cancer stem cells. We investigated the impact of salinomycin on survival, colony growth, migration and invasion of the differentiated human non-small cell lung cancer lines LNM35 and A549. Salinomycin caused concentration- and time-dependent reduction in viability of LNM35 and A549 cells through a caspase 3/7-associated cell death pathway. Similarly, salinomycin (2.5–5 µM for 7 days) significantly decreased the growth of LNM35 and A549 colonies in soft agar. Metastasis is the main cause of death related to lung cancer. In this context, salinomycin induced a time- and concentration-dependent inhibition of cell migration and invasion. We also demonstrated for the first time that salinomycin induced a marked increase in the expression of the pro-apoptotic protein NAG-1 leading to the inhibition of lung cancer cell invasion but not cell survival. These findings identify salinomycin as a promising novel therapeutic agent for lung cancer.

## Introduction

Lung cancer is the most common form of cancer with one of the highest mortality rates in the world. The chemotherapeutic agents currently in use for lung cancer are unsatisfactory due to associated lack of efficacy, drug resistance, and co-lateral toxicity. Targeted therapies for selected subgroups of patients constitute a remarkable progress in the treatment of lung cancer. However, more than 50% of lung cancers do not have any specific genetic profile and are thus not good candidates for targeted therapy. Despite these advances, current treatments of lung cancer can prolong life by months but do not cure the disease [1]; [2]; [3].

Salinomycin is a polyether antibiotic used to kill gram-positive bacteria including mycobacteria, protazoans such as plasmodium falciparum, and the parasites responsible for the poultry disease coccidiosis. It is also commonly fed to ruminant animals to improve nutrient absorption and feed efficiency [4]; [5]. This old agent is now a serious anti-cancer drug candidate [6]; [7]. First, it has been shown that salinomycin selectively inhibit breast tumor stem cells, suggesting that it can be used as an anticancer drug [8]. Salinomycin was also identified as a selective inhibitor of leukemia stem cells, osteosarcoma stem cells as well as pancreatic cancer stem cells [9]; [10]; [11].

It has been reported that a variety of cancer treatments, such as gamma irradiation, cytotoxic drugs, NSAIDs, markedly increase the expression level of the NSAID-activated gene NAG-1 [12]. NAG-1, also known as Macrophage inhibitory cytokine (MIC-1), and growth and differentiation factor-15 (GDF-15), is a member of the transforming growth factor beta (TGF-β) super-family which can mediate the apoptosis induced by non-steroidal anti-inflammatory drugs in cells not expressing cyclooxygenase [13]; [14]. In general, NAG-1 acts as a tumor suppressor protein by inhibiting tumor growth and inducing apoptosis in the early stages of cancer and several studies show NAG1 induction being associated with cell cycle arrest and apoptosis [15].

The current study investigate the impact of salinomycin on survival, colony growth, migration, and invasion of differentiated human non-small cell lung cancer cells LNM35 and A549 and the potential implication of NAG-1 in these effects.

## Materials and Methods

### Cell culture and reagents

Human lung cancer cells LNM35 [16] and A549 were maintained in RPMI 1640 (Invitrogen, Paisley, UK). All media were supplemented with antibiotics (penicillin 50 U/ml; streptomycin 50 µg/ml) (Invitrogen, Cergy Pontoise; France) and with 10% fetal bovine serum (FBS, Biowest, Nouaille; France). Salinomycin was purchased from Sigma-Aldrich (Sigma-Aldrich, Saint-Quentin Fallavier; France).

### Cell viability

Cells were seeded at a density of 5,000 cells/well into 96-well plates. After 24 h, cells were treated for another 24 and 48 h with different concentrations of salinomycin (0.1–50 µM) in triplicate. Control cultures were treated with 0.1% DMSO (the drug vehicle). The effect of salinomycin on cell viability was determined using the CellTiter-Glo Luminescent Cell Viability assay (Promega Corporation, Madison; US), based on quantification of ATP, which signals the presence of metabolically active cells. The luminescent signal was measured using the GLOMAX Luminometer system. Data were presented as proportional viability (%) by comparing the salinomycin-treated cells with the DMSO-treated cells, the viability of which is assumed to be 100%.

### Caspase 3/7 activity

Cells were seeded at the density of 5,000 cells/well into 96-well plate and treated with salinomycin (10 and 50 µM) for 24 h, in triplicate. Control cells were exposed to DMSO at a concentration equivalent to that of the salinomycin-treated cells (0.1% DMSO). Caspase-3/7 activity was measured using a luminescent Caspase-Glo 3/7 assay kit following the manufacturer's instructions (Promega Corporation, Madison, USA). Caspase reagent was added and the plate was mixed and incubated for 2.5 h at room temperature. Luminescence was measured using a GLOMAX Luminometer system.

### Soft-agar colony growth assay

A layer of agar containing 1 ml of 2.4% low melting temperature Noble agar dissolved in distilled water was poured into wells of a 6-well cell culture dish and allowed to set at 4°C for 5 minutes then incubated at 37°C for 30 min. A second layer (2.9 ml) containing 0.3% of low melting Noble agar dissolved in growth media containing cells (5×10^3^ cells/ml) was placed on top of the first layer and allowed to set at 4°C for 5 minutes then transfer them to the humidified incubator at 37°C for 30 minutes to 1 hour. Growth medium (2 ml) was added on top of the second layer and the cells were incubated in a humidified incubator at 37°C for 14 days and then treated for another 7 days with salinomycin (2.5 and 5 µM). Control cells were exposed to 0.1% DMSO. Medium was changed twice a week. At the end of the experiment, colonies were stained for 1 hour with 2% Giemsa stain, and incubated with PBS overnight to remove excess Giemsa stain. The colonies were photographed and scored. Data were presented as proportional colonies (%) by comparing the salinomycin-treated colonies with the DMSO-treated colonies, the colonies of which is assumed to be 100%.

### Wound healing motility assay

LNM35 and A549 cells were grown in six-well tissue culture dishes until confluent. Cultures were incubated for 10 min with Moscona buffer. A scrape was made through the confluent monolayer with a plastic pipette tip of 1 mm diameter. Afterwards, the dishes were washed twice and incubated at 37°C in fresh RPMI containing 10% fetal calf serum in the presence or absence of the non-toxic concentrations of salinomycin (0.1–0.5 µM) for A549 and salinomycin (0.05–0.1 µM) for LNM35 cells. Control cells were exposed to 0.1% DMSO. At the bottom side of each dish, two arbitrary places were marked where the width of the wound was measured with an inverted microscope (objective ×4). Motility was expressed as the mean ± SEM of the difference between the measurements at 0, 6, 24 and 30 h after wound.

### Matrigel invasion assay

The invasiveness of the lung cancer cells LNM35 treated with salinomycin (0.05–0.1 µM) and A549 cells treated with salinomycin (0.1–0.5 µM) were tested using BD Matrigel Invasion Chambers (8-µm pore size; BD Biosciences, Le Pont de Claix, France) according to manufacturer's protocol. Briefly, cells (1×10^5^ cells in 0.5 ml of media and the indicated concentration of salinomycin) were seeded into the upper chambers of the system, the bottom wells in the system were filled with RPMI supplemented with 10% fetal bovine serum as a chemo-attractant and then incubated at 37°C for 24 h. Control cells were exposed to 0.1% DMSO. Non-penetrating cells were removed from the upper surface of the filter with a cotton swab. Cells that have migrated through the Matrigel were fixed with 4% formaldehyde, stained with DAPI and counted in 25 random fields under a microscope. The assay was carried out in duplicate and repeated three times for quantitative analysis. Data were presented as proportional invasiveness (%) by comparing the salinomycin-treated cells with the DMSO-treated cells, the invasion of which is assumed to be 100%.

### PCR amplification for NAG-1 mRNA

Isolation of total RNA from cells was performed using a Maxwell 16 Research System (Promega) with a total RNA purification kit (Promega) according to the manufacturer's instructions. The concentration and purity of the RNA samples were determined by measuring the absorbance at 260 nm (A260) and the ratio of the absorbance at 260 nm and 280 nm (ND-1000, NanoDrop Technologies Inc, Wilmington, DE, USA).

Gene Expression analysis was performed using two steps RT-PCR reaction. Firstly Total RNA was converted into cDNA using a High Capacity cDNA Reverse Transcription Kit (Life Technologies, Integrated Gulf Biosystems, Dubai, UAE #4374966) at a final concentration of 50 ng RNA in a 20 µl PCR reaction with 10× RT Buffer 2.0 µl, 25× dNTP Mix (100 mM) 0.8 µl, 10× RT Random Primers 2.0 µl, MultiScribe™ Reverse Transcriptase 1.0 µl, RNase inhibitor 1.0 µl, Nuclease-free water 3.2 µl. Reverse transcription was carried out on a Veriti thermocycler (Life Technologies), using the following parameters: 25°C for 10 min, 37°C for 120 min, and 85°C for 5 min. Quantitative real-time PCR assay for the gene of interest was performed in triplicate on a Fast ABI Prism 7900HT sequence detection system (Life Technologies) using a predesigned Taqman gene expression assay for NAG-1 (GDF15 Life Technologies #Hs00171132_m1) and the Human Hypoxanthine phosphoribosyl transferase (Hprt1 rRNA) as the endogenous control (Life Technologies #4326321E). From the diluted cDNA, 4 µl (20 ng) was used as a template in a 10 µl singleplex PCR reaction containing 2X TaqMan ® Fast Universal PCR Master Mix, No AmpErase ® UNG (5 µl) (Life Technologies #4352042), 20× TaqMan® Gene Expression Assay (0.5 µl), RNase free water 0.5 µl. The PCR thermal cycling parameters were run in Fast mode as follows 50°C for 2 min, 95°C for 20 s followed by 40 cycles of 95°C for 1 s and 60°C for 20 s. Results were initially analyzed with the ABI Prism 7900HT SDS program v2, 4, all remaining calculations and statistical analysis were performed by the SDS RQ Manager 1.1,4 software using the 2−ΔΔCt method with a relative quantification RQmin/RQmax confidence set at 95%

### Expression of the pro-apoptotic protein NAG-1

LNM35 and A549 cells were seeded in 100 mm dishes at 3×10^6^ cells/dish for 24 h and with increasing concentrations of salinomycin (1–50 µM) for another 24 h. Control cells were exposed to 0.1% DMSO. Total cellular proteins were isolated using RIPA buffer (25 mM Tris.HCl pH 7.6, 1% nonidet P-40, 1% sodium deoxycholate, 0.1% SDS, 0.5% protease inhibitors cocktail, 1% PMSF, 1% phosphatase inhibitor cocktail) from control and treated cells. The whole cell lysate was recovered by centrifugation at 14,000 rpm for 20 minutes at 4°C to remove insoluble material and 30 µg of proteins were separated by SDS-PAGE gel for NAG-1 expression (Cellular Signaling Technology, MA; USA). After electrophoresis, the proteins were transferred on a nitrocellulose membrane, blocked with 5% non-fat milk and probed with NAG-1 (1∶200) and β-actin (1∶1000) antibodies overnight at 4°C. The blot was washed, exposed to secondary antibodies and visualized using the ECL system (Pierce).

### Establishment of stable NAG-1 silencing in lung cancer cells and its impact on the inhibition of cell viability and invasion induced by salinomycin

A549 cells were seeded at a density of 20,000 cells/well into 96-well plates, in the presence of the serum and allowed to attach for 24 h. Cells were transfected with three different designs of SMARTvector 2.0 Lentiviral shRNA particles targeting NAG-1 or SMARTvector 2.0 Non-Targeting control particles (Dharmacon Thermo Scientific, US) that contained a puromycin resistance gene for selection and GFP for identification of positive clones and pools of clones. Selection of cells stably expressing NAG-1 shRNA and the Control shRNA started 72 h post-transfection following the manufacturer's instructions (Dharmacon Thermo Scientific, US). Briefly, growth medium was aspirated from the cells and replaced with fresh selection medium containing 10 µg/mL of puromycin. Puromycin-containing medium was replaced every 2–3days with freshly prepared selection medium, and selection of stable cells expressing NAG1-shRNA or Control shRNA was completed in approximately 4 weeks from commencement of selection. Multiple clones and pools of clones were expanded, and the GFP positive pools of clones were analyzed using western-blot to investigate the expression of NAG-1 following treatment with salinomycin 5 µM. Next, we investigate the invasiveness and the viability of A549 cells expressing NAG1-shRNA or Control shRNA in response to salinomycin.

### Statistics

Results were expressed as mean ± S.E.M. of the number of experiments. The difference between experimental and control values were assessed by ANOVA followed by Dunnett's post-hoc multiple comparison test. P<0.05 indicate a significant difference.

## Results

### Salinomycin inhibits lung cancer cell viability

**Figure 1:**
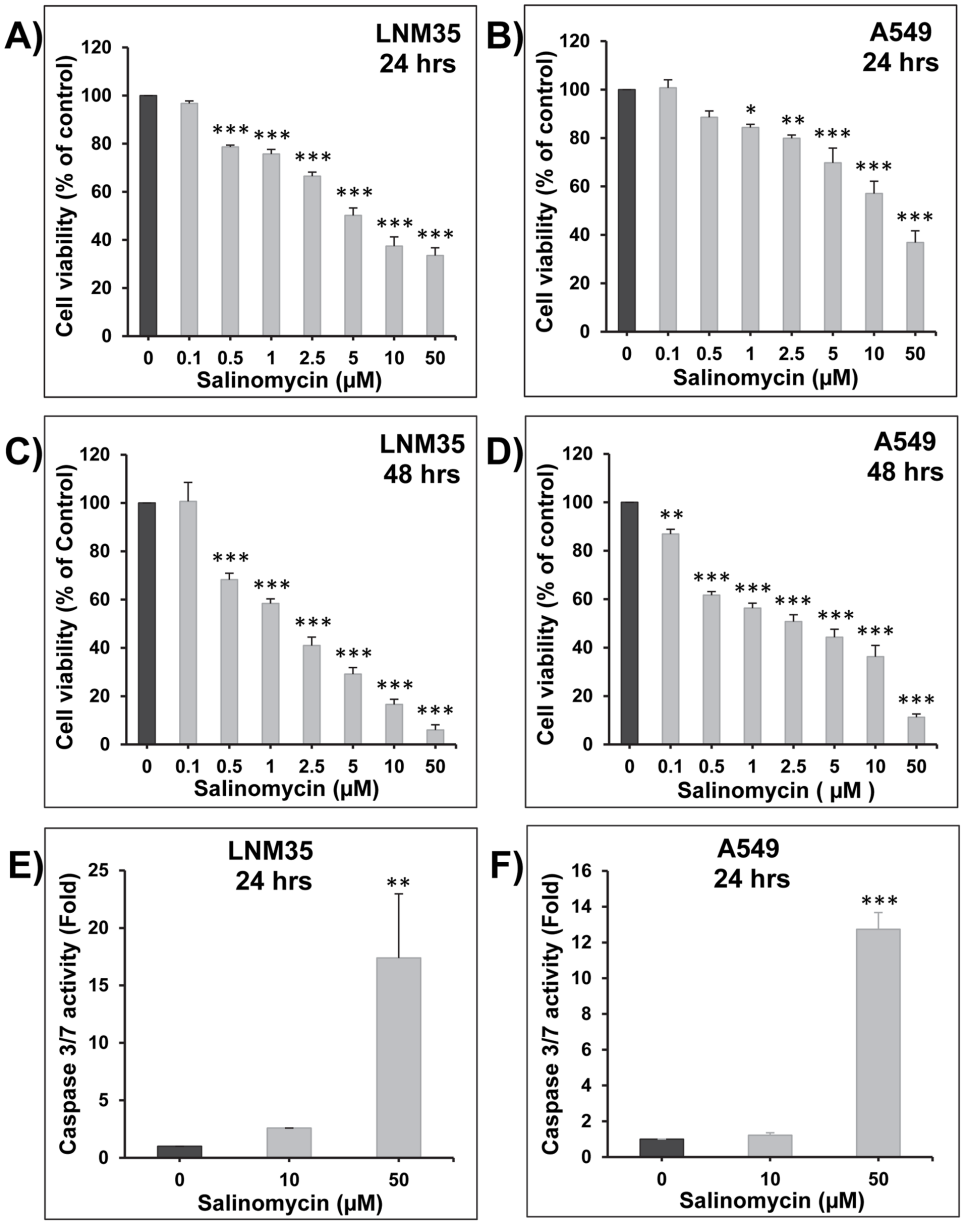
Inhibition of cell viability by salinomycin. Exponentially growing LNM35 (**A, C**) and A549 (**B, D**) cells were treated with vehicle (0.1% DMSO) or the indicated concentrations of salinomycin. The left panel represents 24 h exposure, while the right panel represents 48 h exposure. Viable cells were assayed as described in Materials and Methods. Induction of caspase-3/7 activity was analyzed in LNM35 (**E**) and A549 (**F**) cells treated for 24 h with salinomycin (10 and 50 µM), normalized to the number of viable cells per well and expressed as fold induction compared with the control group. All experiments were repeated at least three times. *Significantly different at P<0.05, **Significantly different at P<0.01, ***Significantly different at P<0.001.

Exposure of LNM35 **(** **Figure. 1A** **)** and A549 **(** **Figure. 1B** **)** cells to salinomycin concentrations (0.1–50 µM) for 24 or 48 h decreased cell viability in concentration- and time-dependent manner. The IC_50_ concentrations at 24 h were in the range of 5 to 10 µM salinomycin for both cell lines. After 48 h treatment, the IC_50_ concentrations decreased to the range of 1.5 to 2.5 µM salinomycin with 90% inhibition of cell viability in both cells at the concentration of 50 µM.

Activation of caspase-3/7 is essential in apoptotic cell death pathways and was analyzed in LNM35 and A549 cells treated for 24 h with salinomycin (10–50 µM), and normalized to the number of cells per well. Caspase 3/7 activity increased by more than 17-fold in LNM35 cells **(** **Figure. 1C** **)** and 12-fold in A549 cells **(** **Figure. 1D** **)**, following exposure to salinomycin 50 µM. This indicates that salinomycin induce cell death through a caspase 3/7-associated pathway.

### Salinomycin impairs colony growth in soft agar

**Figure 2:**
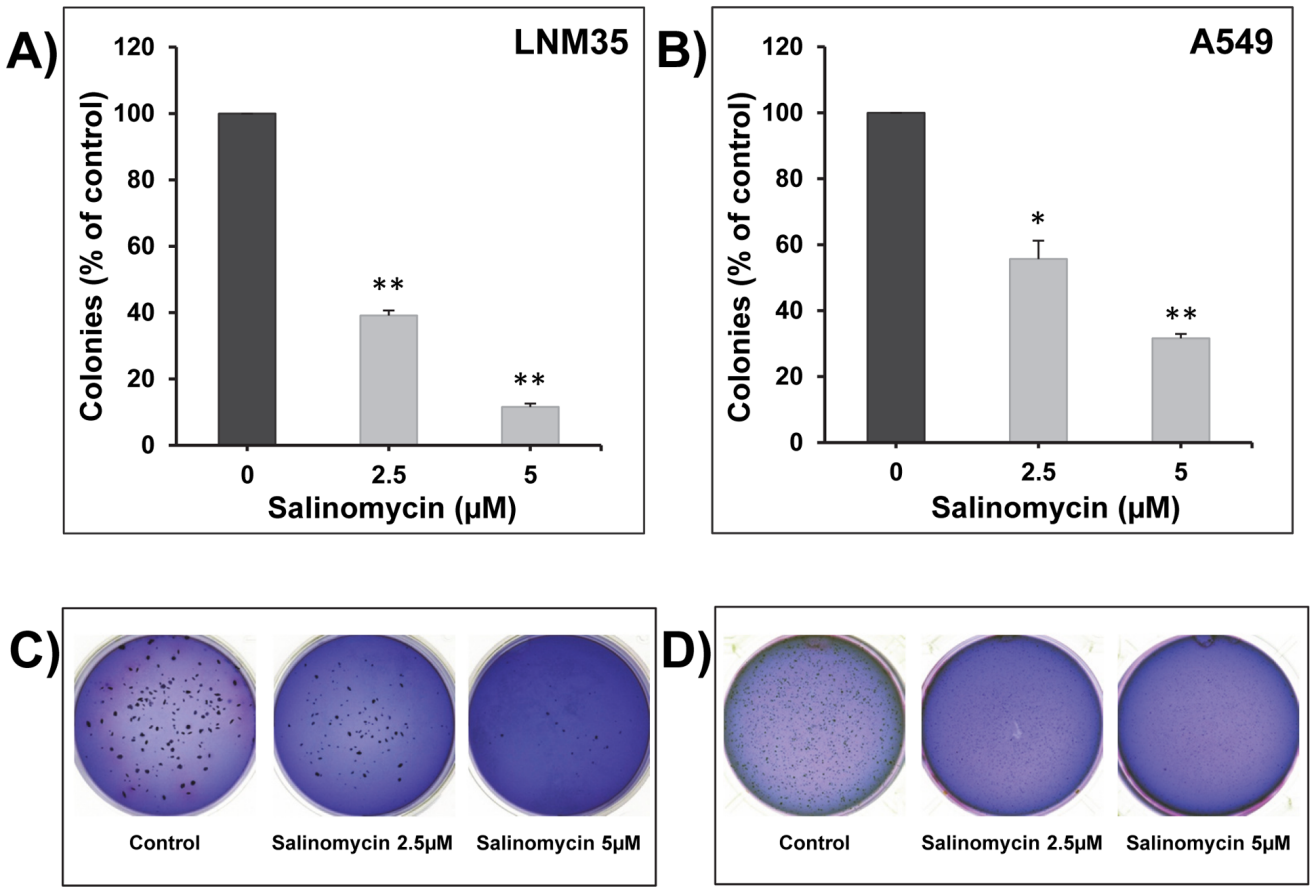
Salinomycin impairs colony growth in soft agar. LNM35 (**A**) and A549 (**B**) cells were grown at a density of 5,000 cells/well in soft agar medium into 6-well plates. After 14 days, formed colonies were treated for 7 days with different concentrations of salinomycin (2.5 and 5 µM). At the end colonies were stained with Giemsa and scored. Representative pictures of the colonies formed in soft agar from LNM35 (**C**) and A549 (**D**) were photographed.

Salinomycin strongly inhibited LNM35 **(** **Figure. 2A, 2C** **)** and A549 **(** **Figure. 2B, 2D** **)** colony growth in soft-agar in a concentration-dependent manner.

### Salinomycin impairs lung cancer cell migration and invasion

**Figure 3:**
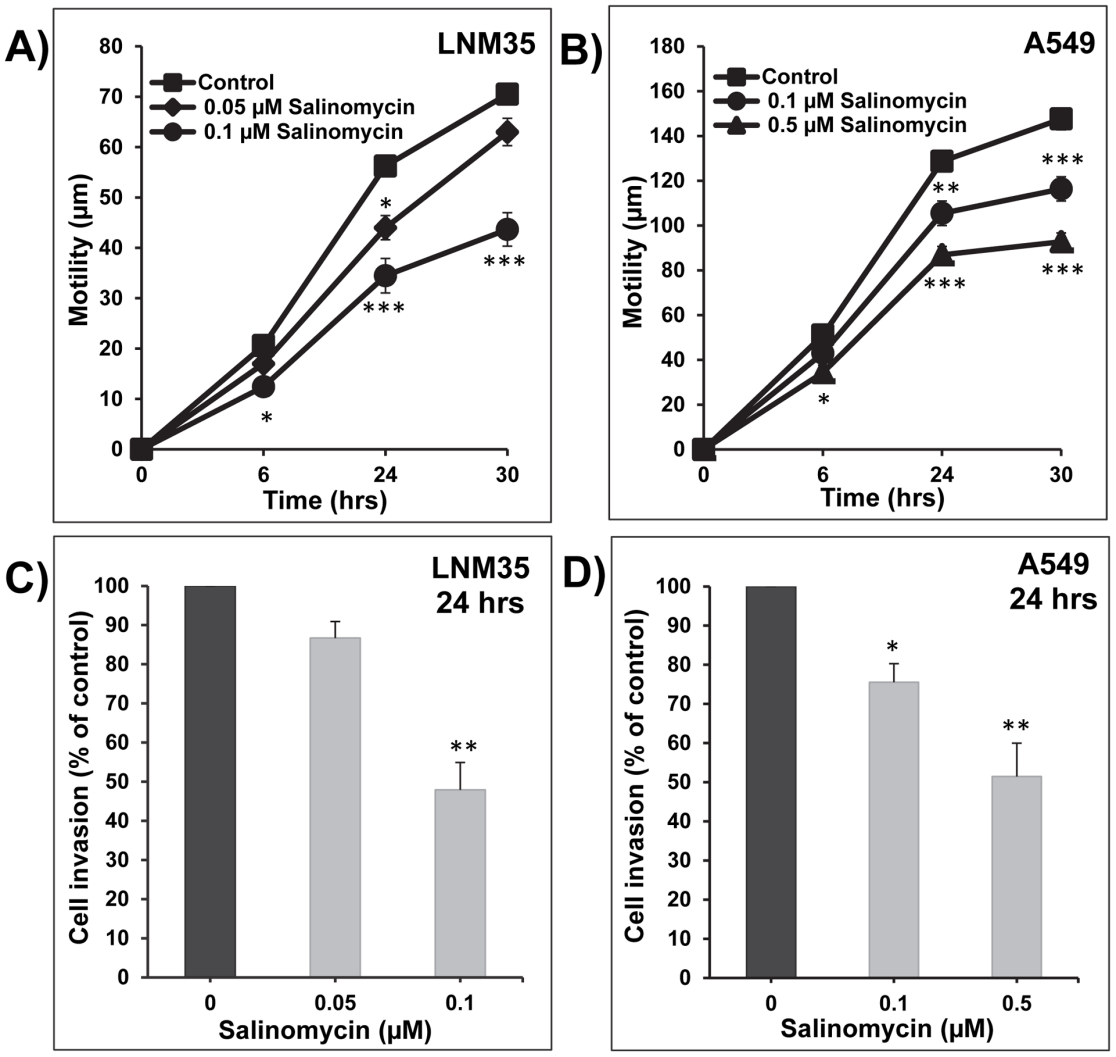
Salinomycin impairs lung cancer cell migration and invasion. Wounds were introduced in LNM35 (**A**) and A549 (**B**) confluent mono-layers cultured in the presence or absence (control) of salinomycin (0.05–0.5 µM). The mean distance that cells travelled from the edge of the scraped area for 6, 24, and 30 h at 37°C was measured in a blinded fashion, using an inverted microscope. **C**) LNM35 cells were incubated for 24 h in the presence or absence of salinomycin (0.05–0.1 µM). **D**) A549 cells were incubated for 24 h in the presence or absence of salinomycin (0.1–0.5 µM). Cells that invaded into Matrigel were scored as described in Materials and Methods.

Lung cancer patients are at high risk of development of metastasis, starting with cell migration in the primary tumor, leading to local tissue invasion and entry into lymph or blood vessels and finally colonization of distant organs. The ability of salinomycin to reduce cellular migration was investigated using wound-healing model. Salinomycin reduced cellular migration of LNM35 **(** **Figure. 3A** **)** and A549 **(** **Figure. 3B** **)** cells in a concentration- and time-dependent manner. Similarly, salinomycin impaired the invasion of LNM35 **(** **Figure. 3C** **)** and A549 **(** **Figure. 3D** **)** cells in the matrigel invasion assay. Salinomycin inhibition of cellular migration and matrigel invasion occurred without significant reduction of cell viability **(** **Figure. 1** **)**.

### Salinomycin induces NAG-1 mRNA and protein expression

**Figure 4:**
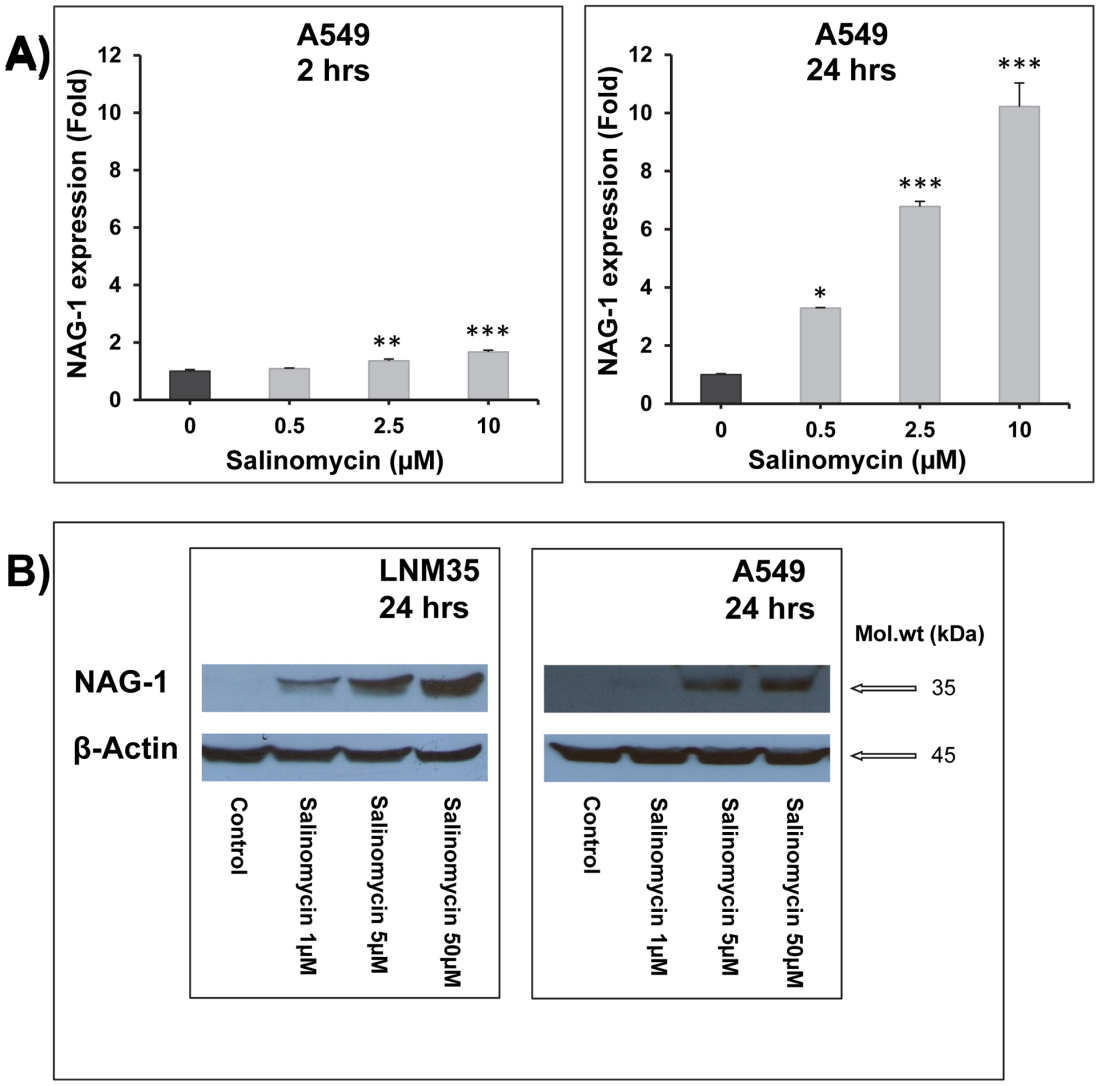
Induction of NAG-1 expression by salinomycin. Cells were exposed to different concentrations of salinomycin, and total RNA was extracted after 2 and 24 hours and analyzed for NAG-1 mRNA expression in A549 cells (**A**) and after 24 h for protein expression in both A549 and LNM35 cells (**B**).

First, A549 cells were exposed to different concentrations of salinomycin (0.5–10 µM) for 2 and 24 h total RNA was extracted and evaluated for NAG-1 mRNA expression. As shown in figure 4A, salinomycin induces a marked time- and concentration-dependent induction of NAG-1 mRNA expression with a ten fold increase reached at 24 h after exposure to 10 µM salinomycin **(** **Figure. 4A** **)**. Next, LNM35 and A549 cells were exposed to different concentrations of salinomycin (1–50 µM) for 24 h and total proteins were evaluated for NAG-1 expression. In both LNM35 and A549 cells, salinomycin induces a marked concentration-dependent induction of NAG-1 protein expression **(** **Figure. 4B** **)**.

### NAG-1 silencing abrogate the anti-invasive effect of salinomycin

**Figure 5:**
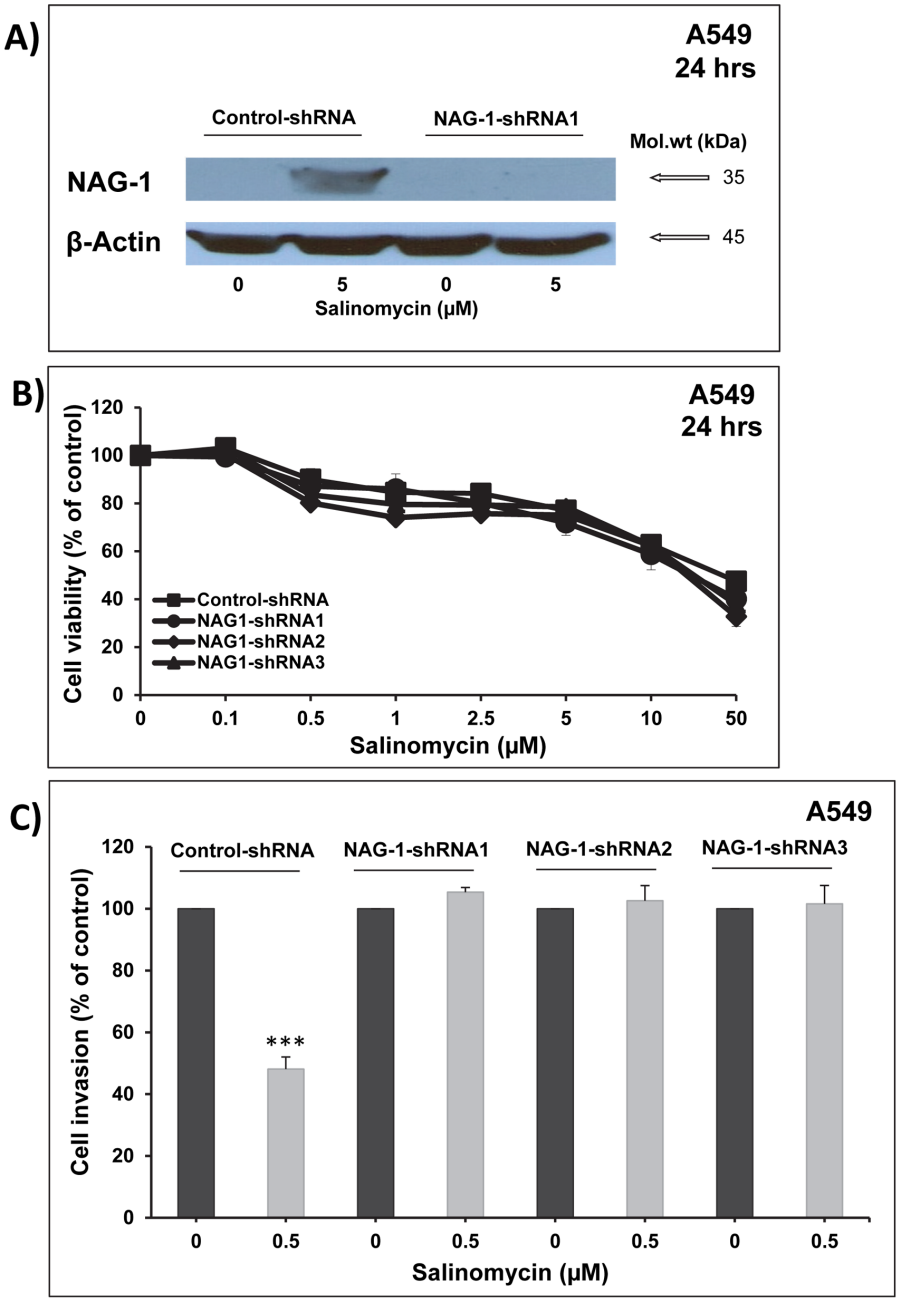
The induction of NAG-1 expression by salinomycin mediate its anti-invasive potential. Silencing of NAG-1 suppressed the increased expression of NAG-1 induced by salinomycin (**A**) without impact on the inhibition of the A549 cell viability induced by salinomycin (**B**) leading to complete suppression on anti-invasive potential of salinomycin (**C**).

As shown in **Figure 5A**, silencing of NAG-1 (NAG-1-shRNA1) reversed the induction of NAG-1 protein expression after treatment with 5 µM of salinomycin. Similar results were obtained with the two other designs of shRNA (NAG-1-shRNA2 and NAG-1-shRNA3) (data not shown). The selectivity of this silencing was confirmed by the fact that no impact on NAG-1 protein expression was observed in the cells transfected with shRNA control particles (control-shRNA) in comparison with the non-transfected cells. Silencing of NAG-1 induction using three different designs of NAG-1-shRNA (1, 2 and 3) failed to decrease the ability of salinomycin to induced A549 cell death **(** **Figure 5B** **)**, but completely reversed the anti-invasive effect of salinomycin **(** **Figure 5C** **)**. All together, these results strongly suggest that NAG-1 mediates the anti-invasive, but not the cell death effect of salinomycin.

## Discussion

Lung cancer is the most common type of cancer worldwide and also has the highest mortality rate. There were 1.61 million cases of lung cancer (12.7% of total cancer burden) in 2008 with 1.38 million deaths (18.2% of all cancer deaths) in the same year [17]. Unfortunately, despite advances in molecular biology of lung cancer, improved diagnosis and even the optimal targeted therapies, the current protocols for the treatment of lung cancer are still insufficient to produce clear and sustained clinical benefits and the cure of lung cancer patients remains unsuccessful. Patients frequently develop resistance to the current targeted therapy drugs and these drugs are also very expensive and not available to the majority of lung cancer patients in the world [18].

In the present study, we investigated the impact of salinomycin on two differentiated human non-small lung cancer cells (LNM35 and A549) survival, colony growth, migration and invasion and the potential role of NAG-1 in the potential anti-cancer effects of salinomycin.

Salinomycin (0.1–50 µM) decreased the viability of LNM35 and A549 differentiated cells in a concentration-, time-dependent and caspase-associated manner. These results are in-line with previously published results showing that low concentrations of salinomycin inhibit the viability of breast cancer stem cells, leukemia stem cells, osteosarcoma stem cells, as well as pancreatic cancer stem cells [8]; [9]; [10]; [11]. During the preparation of this study, another group demonstrated the anticancer activities of salinomycin *in vitro* against the stem cell sub-population in the A549 cells [19]. To confirm this anticancer effect of salinomycin, we investigated its effect on the growth of established colonies. We demonstrated that seven days treatment with low concentration of salinomycin strongly inhibited LNM35 and A549 colony growth in soft agar. These results *in vitro* are in line with other studies demonstrating that salinomycin inhibits breast cancer, osteosarcoma, pancreatic cancer, as well as hepatocellular carcinoma growth in mice [8]; [10]; [11]; [20]. The doses of salinomycin used in these *in vivo* studies were between 4 and 8 mg/kg/I.P which are lower than the LD_50_ dose of salinomycin which was 18 mg/kg intra-peritoneally and 50 mg/kg orally [4]. All together suggest that salinomycin may be a valid and safe anti-cancer agent and further studies will determine the impact of low dose of salinomycin on LNM35 and A549 tumor growth *in vivo* in nude mice.

In the current study, only the highest concentration of salinomycin (50 µM) was able to induce an activation of caspase 3/7 leading to cell death in both lung cancer cells LNM35 and A549. These results are consistent with our recently published work showing that high concentration of salinomycin (50 µM) induced an activation caspase 3/7 and PARP cleavage leading to apoptosis of the breast cancer cells MDA-MB-231 [21]. However, both studies on lung cancer cells and the previously published study on breast cancer cells shows that low concentrations of salinomycin (≤10 µM) resulted more in inhibition of cell proliferation rather than cell death [21]. Similarly, it has been demonstrated that salinomycin inhibits proliferation and incudes apoptosis of human hepatocellular carcinoma [20].

Cancer progression is associated with abrogation of the normal controls that limits cell migration and invasion, eventually leading to metastasis. Since metastases are the major cause of death in cancer patients, the development of new treatment regimens that reduce invasion and metastasis is highly important for cancer therapy. In this context, we demonstrated that salinomycin induced a highly significant time- and concentration-dependent inhibition of cell migration and invasion *in vitro* of the two differentiated lung cancer cell lines LNM35 and A549. These results are in agreement with previous studies showing that salinomycin selectively inhibits breast cancer stem cells metastases *in vivo* [8] and prostate cancer cell migration *in vitro* [22].

The anti-cancer molecular mechanism of action of salinomycin has been investigated in several studies. In this context, it has been demonstrated that salinomycin is an effective anticancer agent that induced apoptosis in human cancer cells independent of tumor suppressor protein p53 and caspases activation [9]. These results are in agreement with our recent study showing that salinomycin significantly decreased viability of the wild-type p53 MCF-7 and T47D as well as mutant p53 MDA-MB-231 and T47D human breast cancer cell lines in time- and concentration-dependent manners [21]. It was also shown that salinomycin triggers apoptosis of PC3 prostate cancer cells by elevating the intracellular ROS level, leading to the translocation of Bax protein to mitochondria, cytochrome c release, activation of the caspase-3, and cleavage of PARP [23]. Salinomycin also induces caspase dependent cell-death in RKO, SW480 and SW620 colon cancer cells [24]. It has also been demonstrated that salinomycin is an effective inhibitor of osteosarcoma stem cells as well as lymphocytic leukemia cells partially through down-regulation of the Wnt/µ-catenin self-renewal pathway [10]; [20]; [25]. Another study showed that salinomycin inhibited prostate cancer cell growth by reducing aldehyde dehydrogenase and nuclear factor-µB activities [22]. Similarly, it has been demonstrated that salinomycin inhibits Akt/NF-κB pathway leading to apoptosis in cisplatin resistant ovarian cancer cells [26]. It has also been demonstrated that salinomycin induces autophagy in colon and breast cancer cells [24]. Increased phosphorylation of the DNA damage-related proteins (e.g. pH2AX, p53BP1, pBRCA1, pChk1) proteins indicates greater DNA breakage and damage [27]. Salinomycin increases DNA strand breaks and induces phosphorylation of the DNA damage-related protein, H2AX [28].

The NSAID-activated gene (NAG-1, GDF-15, and MIC-1) is induced by several apoptosis-inducing agents in colon, prostate, and lung cancer cells [15]; [29]. NAG-1 was involved in the synergistic induction of apoptosis by combined treatment of sodium salicylate and PI3K/MEK1/2 inhibitors in A549 human lung adenocarcinoma cells [30]. The NAG-1 expression levels substantially increase in cancer cells, serum, and/or cerebrospinal fluid during the progression of diverse human aggressive cancers, such as intracranial brain tumors, melanoma, gastrointestinal, pancreatic, colorectal, prostate, breast, and lung epithelial cancers. Of clinical interest, an enhanced NAG-1 expression has been positively correlated with poor prognosis and patient survival [29]; [30]; [31]; [32].

We hypothesized that NAG-1 is involved in the anti-cancer effects of salinomycin and we demonstrated for the first time that salinomycin induced a clear time- and concentration-dependent induction of NAG-1 expression. We also clearly demonstrated that NAG-1 contributes to the inhibition of lung cancer cell invasion but not to the induction of cell death, mediated by salinomycin. In contrast, it has been reported that NAG-1 expression was associated with a more invasive gastric cancer cell line phenotype and could induce increased gastric cancer cell invasion *in vitro* [33].

It has been demonstrated that salinomycin improve the efficacy of gemcitabine to eradicate pancreatic cancer [11]. Salinomycin also sensitizes breast cancer cells to the effects of doxorubicin and etoposide treatment by increasing DNA damage [28]. In the same context, the combination therapy of octreotide modified paclitaxel active targeting micelles and salinomycin passive targeting micelles improve the treatment of breast cancers through the eradication of breast cancer cells together with breast cancer stem cells [34]. Similarly, the combination therapy of trastuzumab and salinomycin was superior to single treatment with each drug in the treatment of HER2-positive breast cancer cells as well as breast cancer stem cells [35]. We recently reported *in vitro*, that salinomycin was also able to potentiate the anticancer effects of the 4-Hydroxytamoxifen and the frondoside A on breast cancer cells MCF-7 and MDA-MB-231 respectively [21]. Building on these aforementioned results, and knowing from clinical trials that single agent treatments rarely result in clinical benefits to cancer patients, and that combination therapies are in usual necessary for effective treatment of tumors, we investigated the therapeutic advantage of combination of cisplatin, (a first line therapeutic for lung cancer), with salinomycin in both LNM35 and A549 cells. Unfortunately, we found that cisplatin was not able to enhance the inhibition of lung tumor cell viability induced by salinomycin (Attoub et al, Unpublished data).

The current finding identifies salinomycin as a promising novel therapeutic agent not only for the depletion of cancer stem cells but also for the differentiated lung tumors.
